# The Grayness of the Origin of Life

**DOI:** 10.3390/life11060498

**Published:** 2021-05-29

**Authors:** Hillary H. Smith, Andrew S. Hyde, Danielle N. Simkus, Eric Libby, Sarah E. Maurer, Heather V. Graham, Christopher P. Kempes, Barbara Sherwood Lollar, Luoth Chou, Andrew D. Ellington, G. Matthew Fricke, Peter R. Girguis, Natalie M. Grefenstette, Chad I. Pozarycki, Christopher H. House, Sarah Stewart Johnson

**Affiliations:** 1 Department of Geosciences, The Pennsylvania State University, University Park, PA 16802, USA; akh5390@psu.edu; 2 Earth and Environmental Systems Institute, The Pennsylvania State University, University Park, PA 16802, USA; 3 NASA Goddard Space Flight Center, Greenbelt, MD 20771, USA; danielle.n.simkus@nasa.gov (D.N.S.); heather.v.graham@nasa.gov (H.V.G.); luoth.chou@nasa.gov (L.C.); chad.i.pozarycki@nasa.gov (C.I.P.); 4 NASA Postdoctoral Program, USRA, Columbia, MD 20146, USA; 5 Department of Physics, Catholic University of America, Washington, DC 20064, USA; 6 Santa Fe Institute, Santa Fe, NM 87501, USA; elibbyscience@gmail.com (E.L.); ckempes@santafe.edu (C.P.K.); natalie.grefenstette@santafe.edu (N.M.G.); 7 Department of Mathematics and Mathematical Statistics, Umeå University, 90187 Umeå, Sweden; 8 Icelab, Umeå University, 90187 Umeå, Sweden; 9 Department of Chemistry and Biochemistry, Central Connecticut State University, New Britain, CT 06050, USA; smaurer@ccsu.edu; 10 Department of Earth Sciences, University of Toronto, Toronto, ON M5S 3B1, Canada; barbara.sherwoodlollar@utoronto.ca; 11 Department of Biology, Georgetown University, Washington, DC 20057, USA; 12 Department of Molecular Biosciences, College of Natural Sciences, The University of Texas at Austin, Austin, TX 78712, USA; ellingtonlab@gmail.com; 13 Center for Systems and Synthetic Biology, The University of Texas at Austin, Austin, TX 78712, USA; 14 Department of Computer Science, University of New Mexico, Albuquerque, NM 87108, USA; mfricke@unm.edu; 15 Department of Organismic and Evolutionary Biology, Harvard University, Cambridge, MA 02138, USA; pgirguis@oeb.harvard.edu; 16 Blue Marble Space Institute of Science, Seattle, WA 98104, USA; 17 Science, Technology and International Affairs Program, Georgetown University, Washington, DC 20057, USA

**Keywords:** origin of life, prebiotic evolution, meteoritic organics, pre-RNAs, metalloenzymes, thioesters, lipids, evolutionary transitions, agnostic biosignatures

## Abstract

In the search for life beyond Earth, distinguishing the living from the non-living is paramount. However, this distinction is often elusive, as the origin of life is likely a stepwise evolutionary process, not a singular event. Regardless of the favored origin of life model, an inherent “grayness” blurs the theorized threshold defining life. Here, we explore the ambiguities between the biotic and the abiotic at the origin of life. The role of grayness extends into later transitions as well. By recognizing the limitations posed by grayness, life detection researchers will be better able to develop methods sensitive to prebiotic chemical systems and life with alternative biochemistries.

## 1. Introduction

In Earth’s history, the specifics of the progressive transition from a non-living world to a living world are unknown, and the distinction between abiotic and biotic systems is often unclear. The origins of life and astrobiology communities have traditionally looked for a metric that clearly distinguishes between non-life and life; however, a single unambiguous signal of life has proven elusive [1,2]. Even if found, such a signal would likely exclude life early in its evolution. A more inclusive representation is to recognize a spectrum between the non-living and the living—a “grayness” resulting from the protracted evolutionary process that gave rise to life [3]. This stepwise progression from abiotic to biotic factors has been problematic in the past because it renders any specific definition of life incapable of specifying a precise threshold for the transition from abiotic to biotic systems. As a result, we have been left with broad conceptions of life or a list of observed traits [3,4,5,6,7].

It should be no surprise that this grayness exists given that the laws governing physics apply equally to geology, chemistry, and biology. Whether inside or outside of a living system, individual chemical reactions progress based on universal, causal rules, and within a huge and rich combinatorial space. Furthermore, life evolves within a planetary context, albeit at a molecular scale, and its biochemistry is dependent on what is permitted by the geochemical environment [8,9]. The grayness inherent in the stepwise transition from geochemistry to biochemistry presents a major obstacle for accurately identifying life in its earliest forms. Interestingly, recent phylogenetic reconstructions of the physiological capabilities of the Last Universal Common Ancestor (LUCA) portray it as only “half alive,” lacking key steps involved in modern biochemical pathways and heavily reliant on geochemical reactions in its environment to provide substrates [10].

During the transition from non-living to living, a number of supporting chemical systems would need to evolve, at least in a primitive form, in order to create and sustain life as we know it. Of these, there is a diversity of ideas about which of these systems were most vital to the evolution of life. Indeed, there is widespread debate about what features and properties actually define life (e.g., [11]), and it is important to recognize that existing frameworks are likely to be subsumed by more general theories in the future. However, individual frameworks provide a lens for us to consider the key concepts of early transitions. One such perspective is the minimum model of life proposed by Tibor Gánti [12], called the chemoton. It is based on the observed features of lipid encapsulation, enzyme catalysis, and genetic material. They were described by Gánti as:

1. The membrane subsystem, which forms autocatalytically and provides compartmentalization;
2. The metabolic subsystem, which transforms external nutrients into materials for internal processes;
3. The information subsystem, which is comprised of a templating polymer that governs the operations of the supersystem.

**Figure 1:**
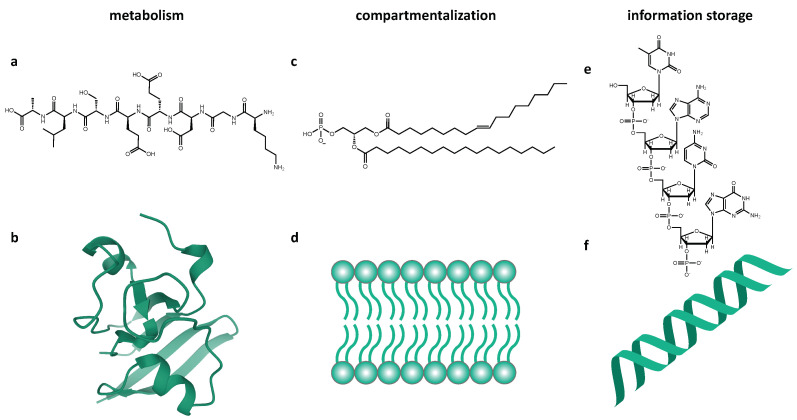
Illustration of the three macromolecules used by life on Earth, as both constituent parts (**a**,**c**,**e**) and larger-scale structures (**b**,**d**,**f**), which fulfill the roles described in the chemoton model: metabolism, compartmentalization, and information storage. (**a**) A polypeptide consisting of eight amino acids. (**b**) Spinach ferredoxin protein structure, PDB 1A70, showing alpha-helices, beta-pleated sheets and loops structures [19]. (**c**) A phospholipid containing fatty acid tails made of repeating 2C units. (**d**) A segment of a phospholipid bilayer, which forms cell membranes. (**e**) A short chain of DNA, illustrating the Watson–Crick nitrogenous bases. (**f**) An A-form double helix showing the structure of DNA storage.

The macromolecules used by life on Earth for biochemistry and their corresponding subsystem are shown in Figure 1. The fact that Gánti’s chemoton does not specify the biochemistry of these subsystems lends it to an agnostic or universal model of life, where alternative molecules could carry out these roles [13].

Grayness would have existed during the development and co-evolution of the subsystems. Once the subsystems were unified, through stoichiometrically coupled cycles, it became the unambiguously living chemoton. Special attention has been given to subsystem integration, both in philosophical and chemical investigations of the origin of life [14,15]. Recent computational work predicting new prebiotic synthesis pathways and primitive reaction networks with self-regenerating cycles hints at how such subsystems may have evolved [16].

The ability to clearly distinguish between abiotic and biotic systems is considered by many a prerequisite for effective astrobiology studies. However, since no natural demarcation truly exists, there is a profound difficulty in advancing the field if we rely on a strict dichotomy. Even definitively identifying abiotic versus biotic sources for a simple organic compound such as methane have proven challenging [17]. A reasonable solution is to allow for partial membership in each classification, with only end-members, typifying the abiotic and biotic, being clearly defined [18]. For the purposes of this discussion, we will define the unified subsystems in Gánti’s chemoton model as the end-member of the biotic class. The multivalued logic approach of partial membership also allows the examination of particular aspects of the living system, depending on how the end-members are defined [18]. As there are likely no intrinsic thresholds, we are left with scales of comparison.

By understanding the inherent grayness of life on Earth, we are better able to develop techniques for recognizing life and identifying prebiotic potential elsewhere. Here, we illustrate how possible earliest steps in the transition towards life are filled with grayness. In fact, grayness is a hallmark of evolution; similar ambiguity has persisted throughout all major life transitions. We focus on some of the most common considerations of life and trajectories from the abiotic to biotic, including possible intermediate forms, and in each case illustrate that grayness exists. We also recognize that there are many general and specific perspectives not covered in our analysis, which is aimed at depicting the ubiquity of grayness rather than exhaustively covering theories of life.

## 2. Organic Molecules

Without the ability to sample early Earth environments, our understanding of Earth’s prebiotic organic chemistry relies heavily on theory and experimental simulations of abiotic organic synthesis. Some extreme Earth systems have been identified where abiotic organic synthesis plays an important role, such as marine hydrothermal vents [20,21] and million to billion year residence fracture fluids isolated in Precambrian rocks kilometers below the surface [22]. While such environments yield insights into the type of non-biological water–rock reactions that would have dominated a prebiotic Earth, even in these extreme environments the influence of biologically driven reactions cannot be ruled out. Extant and extinct biological processes have left, and continue to leave their biological signatures within and beyond Earth’s biosphere. This includes biological alteration of inorganic matter such as minerals as well as residual lipids and other biomolecules that prevent us from easily detecting the *de novo* production of biological precursors in nature. In contrast, meteorites, micrometeorites, and interplanetary dust particles (fragments of asteroids, comets, and other planetary bodies, hereinafter collectively referred to as “meteorites”) serve as examples of end-members on the abiotic-to-biotic spectrum; they are almost certainly devoid of intrinsic biotic components and are amenable to sampling and laboratory analyses. Investigating the composition of meteorites informs us on the distribution of prebiotic organic matter in abiotic systems and the expected chirality of organic compounds of abiotic origins. Furthermore, as frequent meteoritic and cometary impacts may have served as a significant source of exogenous organic matter to the early Earth, the composition of extraterrestrial materials may have played a key role in influencing Earth’s prebiotic chemistry.

**Figure 2:**
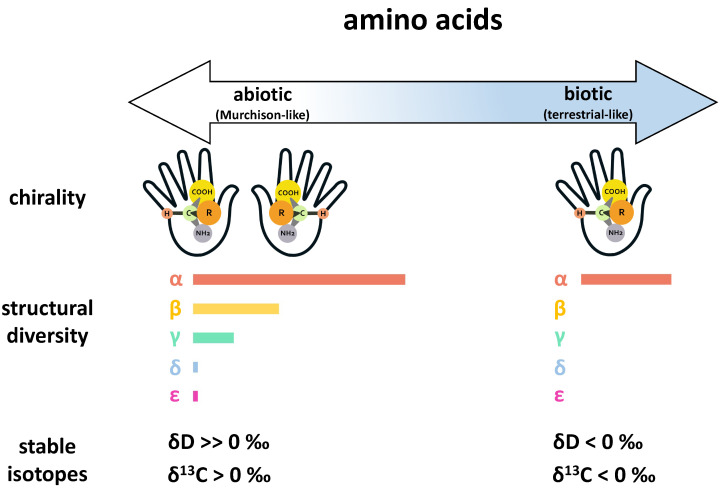
An illustration of amino acid chirality, amino acid structural diversity, and amino acid stable isotope compositions (δD, the isotopic ratio of D/H in the sample relative to Standard Mean Ocean Water (SMOW), an isotopic standard for water, and $\delta^{13}$C, the isotopic ratio of ${}^{13}$C/${}^{12}$C in the sample relative to Pee Dee Belemnite (PDB) standard for carbon) across an abiotic-to-biotic spectrum. Here, the abiotic and biotic end-members are defined by the Murchison (CM2) carbonaceous chondrite (a structurally diverse and well characterized abiotic reference) and terrestrial biology, respectively. **Chirality**: Amino acids of abiotic origins are often racemic or near-racemic, while amino acids in terrestrial biology exhibit distinct homochirality. **Structural diversity:** Amino acids are categorized by the position of the amine group relative to the acid group in the chemical structure (i.e., whether the amine group is positioned at the α, β, γ, δ, or ϵ carbon). For the abiotic end-member, the lengths of the bars represent the number of aliphatic amino acids identified to date in Murchison for each specific category (see Glavin et al., 2018 [23] and references therein). For the biotic end-member, the single bar represents the simple distribution of 20 proteinogenic α-amino acids. **Stable isotopes:** While δD and $\delta^{13}$C values can vary widely depending on the source and history of the sample, meteoritic amino acids of extraterrestrial origins generally exhibit highly positive δD values and often have $\delta^{13}$C values above 0‰ (see Elsila et al., 2012 [36] and references therein). In contrast, amino acids of terrestrial origins generally have δD and $\delta^{13}$C values that fall below 0‰.

While small biologically relevant organic compounds are common to both meteorites and terrestrial biology, there are some key differences in composition attributed to abiotic and biotic origins. For instance, while the building blocks of proteins in terrestrial biology primarily consist of a simple suite of 20 α-amino acids, a much wider diversity of amino acids has been detected in meteorites, with over 90 unique structures that have been named to date, including α-, β-, γ-, and ϵ-amino acids, the majority of which are absent or rare in terrestrial biology [23]. Likewise, distinct structural signatures are often observed for abiotic and biotic chiral molecules (i.e., molecules that have one or more pairs of mirror image structural isomers known as “enantiomers”). For example, intrinsic chiral amino acids in meteorites tend to be racemic or nearly racemic (generally small L-enantiomeric excesses < 20%) [24,25,26,27,28], while amino acids in terrestrial biology generally exhibit homochirality. Terrestrial biology is primarily based on enantiopure L-amino acids, though enantiopure D-amino acids can also play a central biological role (e.g., antibacterial functionality of D-amino acids in bacteria [29,30]). Similarly, terrestrial biology primarily uses D-sugars (e.g., as the chemical building blocks of RNA and DNA). Although the chirality of confirmed extraterrestrial sugars has not yet been established [31], some sugar acids in meteorites have been shown to exhibit large D-enantiomeric excesses [32]. In comparison to amino acids, the distinction between abiotic and biotic sugars is not as well established, and other organic compound classes detected in meteorites exhibit similar grayness. Furthermore, assessing the chirality of abiotic versus biotic sugars is complicated by the presence of more than one carbon center in the molecules. Nevertheless, any compositional and enantiomeric differences that have been observed have allowed us to progress in defining end-members on the abiotic-to-biotic spectrum and establish criteria for the search for life beyond Earth based on thresholds. Figure 2 illustrates some of these differences for amino acids on an abiotic-to-biotic spectrum, using Murchison-like and terrestrial-like end-members.

Although analyses of meteoritic organics have advanced our understanding of the abiotic end-member compositions for small organic molecules and have provided context for chemistry shared between meteorites and terrestrial biology, the process and timeline for the transition between the two end-member compositions remains largely unresolved. It is thought that a natural imbalance of enantiomers, generated on the early Earth or inherited from exogenous origins, may have led to the apparent “selection” of enantiopure amino acids and sugars by terrestrial biology. Given the equivalence of the chemical and physical properties of enantiomers, when chiral molecules are synthesized in the laboratory without an enantioselective driving force, the reaction products are naturally racemic; however, a small initial imbalance of enantiomers can be amplified by abiotic chiral selectivity (e.g., via autocatalytic pathways, reactions mediated by chiral catalysts, polymerization and epimerization, or in association with phase transitions) [29,33,34]. Despite several potential drivers for abiotic amplification of enantio enrichments, chiral molecules can naturally racemize over short geologic timescales; racemization half-lives for some proteinogenic amino acids in aqueous solutions range between 10${}^{3}$ and 10${}^{4}$ years at ambient temperature [35]. As such, it is difficult to assess the timeline of the shift across the abiotic-to-biotic spectrum, considering the unknowns regarding the synthesis and stability of enantiopure systems in early Earth environments. Depending on the mechanisms that led to the emergence of these compositional and chiral signatures in terrestrial biology, for some period during the emergence of life on Earth, the chemistry may have fallen mid-spectrum.

The grayness associated with differentiating abiotic and biotic organic chemistry is important to consider when establishing life detection criteria. Enantiopure biologically relevant organic molecules such as amino acids and sugars are probable indicators for a biological source (terrestrial or extraterrestrial), and highly positive isotopic compositions (e.g., $\delta^{13}C>0$‰, δD≫0‰) are considered indicative of abiotic extraterrestrial origins. However, the origins of large enantiomeric excesses for non-proteinogenic amino acids are not well understood. Likewise, near-zero or negative stable isotope compositions (e.g., $\delta^{13}C\leq 0$‰) are not necessarily indicative of biological contamination. Consequently, deciphering these types of ambiguous organic signatures to assign an abiotic or biotic origin requires investigating more complex organic chemistry in the search for more distinctive biosignatures.

## 3. Information Storage Systems

Although there are a number of competing theories regarding the origin of life, each faces the problem of grayness. One common view of the advent of life is based on the primacy of genetic information storage and transfer. In the chemoton model, this approach would correspond to the evolution of the information subsystem. As one of several examples, the “RNA world” hypothesis is often invoked as a solution to the perceived difficulty of co-evolving DNA and proteins in a prebiotic system, as RNA is capable of performing the dual roles of genetic information storage and catalysis [37]. Within the frame of this hypothesis, RNA is proposed as the first informational storage molecule.

As discussed previously, the early Earth received substantial exogenic delivery of volatiles. Hydrogen cyanide (HCN) and its polymers are ubiquitous, known to be significant components of comets, meteorites, some moon and planetary atmospheres, and the interstellar medium, which suggests HCN polymers were likely some of the first large molecules on Earth [38]. Adenine, a nucleobase found in the genetic systems of DNA and RNA as well as in the energy molecule ATP, is formed abiotically as a HCN pentamer. Additionally, polyamidines, formed from HCN polymers, are predicted to convert to polypeptides in water [38]. In these ways, HCN polymers connect abiotic cosmochemistry to modern biotic information and metabolic subsystems. The “HCN world” describes formation mechanisms of RNA precursors [15,39], as well as other pre-RNA molecules, that would initially be indistinguishable from the abiotic geochemistry of the environment. Furthermore, even though the rich chemistry produced from HCN and other molecules potentially present on the early Earth is referred to as a solution to the prebiotic origin of RNA in a chemical system, it also poses the obvious problem of the need for selection among the complex mixture. Prebiotic RNA synthesis is actively being researched, and some potential solutions have been found, such as phosphorylation of uridine monophosphates under hot evaporating pool conditions [40]. However, as RNA building blocks require several specific steps in their formation, they are often seen as too complex to form the basis of a first informational polymer.

**Figure 3:**
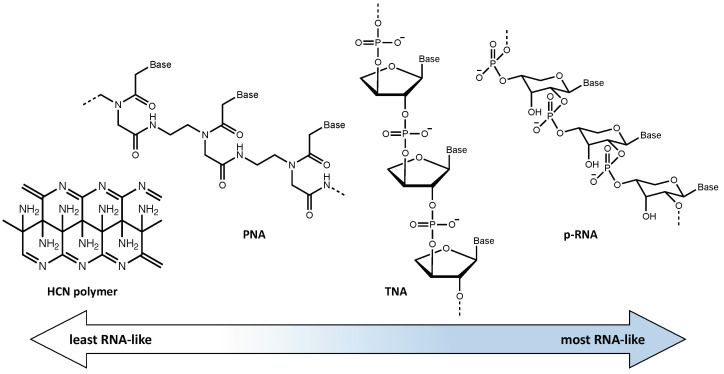
Proposed pre-RNA molecular information systems organized from least to most similar to RNA. From left to right: (1) HCN polymer, (2) peptide nucleic acid (PNA), using an amino acid backbone, (3) threose nucleic acid (TNA), which uses the simpler 4C sugar, and (4) p-RNA which uses the pyranose form of ribose.

In an attempt to avoid this problem, various chemical systems based on pre-RNAs, which fall in an intermediate position on the abiotic-to-biotic spectrum, have been proposed in an attempt to explain the transition to an RNA world. The potential role of pre-RNA is based on the idea that RNA could have been preceded by the evolution of a functionally analogous but simpler prebiotic genetic material. Imagining these pre-RNAs allows us to consider possible alternative genetic systems that may have evolved on early Earth or elsewhere. If a genetic takeover occurred once before, as when DNA became the dominant system over RNA, it is not inconceivable that a similar takeover may have happened other times as well. One, or several, pre-RNAs may have been used by Earth proto-life as part of the transition to the modern biotic system [41]. Although there is much uncertainty regarding what potential pre-RNA(s) may have been present throughout Earth’s history, it is clear that, among the many possibilities described in the literature, there is a range between those more closely resembling abiotic chemistry and those more similar to modern biochemistry. In Figure 3 some of these potential pre-RNAs are shown and their relative similarity to RNA is indicated.

Peptide nucleic acid (PNA) is a potential pre-RNA that does not contain sugar or phosphate, but instead contains a peptide backbone and nucleobases attached via carbonyl methylene linkers [42]. Its structure is achiral, removing the issue of enantiomeric cross inhibition [42,43]. However, it does experience induced chirality when forming a duplex in response to interaction with a chiral amino acid or RNA [43]. It also follows traditional Watson–Crick base pairing rules [42]. Notably, recent work shows N-heterocycles synthesized via electric discharge in neutral and reducing atmospheres form carbonyl side chains, lending support to the possible use of PNA in prebiotic systems [44].PNA does not have a charged backbone, a feature that aids in solubility and discourages folding, however, it is able to grow longer than other uncharged RNA analogs [45]. Interestingly, PNA-based life could to some extent be distinguished based on a C:N ratio, which has limited variability in peptides, but significant grayness would persist due to known abiotic synthesis pathways of its components.

Other potential pre-RNAs arose from Eschenmoser’s project on the chemical etiology of RNA. Eschenmoser’s study was a systematic approach that looked at alternative structures that could fulfill the same role as RNA in a genetic code and that were also potential evolutionary competitors [46]. Potential pre-RNAs resulting from this work were the commonly cited p-RNA (the pyranosyl isomer of RNA) and the promising TNA (α-l-threose nucleic acid) [46,47]. TNA is composed of the four carbon sugar, threose, along with traditional phosphodiester bonds [47]. The simpler structure, easier formation of threose, and increased resistance to hydrolytic cleavage at phosphodiester linkages, in comparison to ribose, make TNA a favorable pre-RNA candidate [47]. Features such as TNA’s canonical Watson–Crick base pairing, and its ability to form a duplex with RNA offer potential mechanisms for the genetic takeover [47]. Only a few of the myriad potential pre-RNA molecules have been described here. Recent computational studies of the chemical space for stable structures compatible with nucleobase genetic information storage show an immense number of potential analogs, including over 200 possibilities for RNA isomers, with chemical formula BC_5_H_9_O_4_, where B is a nucleobase [48,49]. Many of these analogs exist in a chemical space that has largely been unexplored [49]. It is worth noting that when chemical compounds become larger, there is also an increase in the number of structural isomers. This may contribute to grayness in life detection by making a stoichiometric signal harder to detect. Studies probing the abiotic versus biotic controls on isomeric distributions for simple organic molecules such as propane are only beginning to emerge [50].

While N-heterocycles have featured prominently in many of the pre-RNA molecules previously discussed, some even consider the use of a nucleobase genetic code too advanced for the earliest proto-life [51]. Cairns-Smith proposed a primitive gene completely divorced from the concept of biochemistry as we know it, and described a type of pre-RNA that could begin entirely within the inorganic geochemical realm [51]. Cairns-Smith considers nucleic acids a sophisticated product of Darwinian evolution and therefore rejects them as the first information system [51]. Instead, he suggests crystal lattice imperfections as means of storing information and providing a templating surface for replication [51]. This information storage potential was particularly notable in clays because of the phyllosilicate structure, which would also support the later translation into an organic system [51].

The earliest possibilities in the stepwise evolution of information subsystem molecules are the grayest, by definition, posing a considerable challenge to identification in a proto-biotic system. While arising later, more complex molecules that fill this informational role are more easily distinguishable from the abiotic environment by structure and stoichiometry. However, this chemical space becomes increasingly large with this complexity and non-biological isomers may confuse detection.

Another concept of grayness that exists in these information storage molecules is the information content. There was a transition from low information content, in the earliest iterations, to high information content, as is seen in modern biology. This transition may be due to limitations of the system. For instance clays are identified as containing lower information content, in part due to their simplistic storage mechanism [52].

Even in the event that RNA was the first proto-biological information storage molecule, with no widespread system of pre-RNAs before it, there would have been a transition from low functional information to high. The first RNA polymers would have been short oligonucleotides, with length limiting the total information content. Additionally, they would be randomly formed. Randomly formed strings, while having high entropic information, are unlikely to contain evolutionarily useful information. Therefore, the ability to store useful information is determined by the length of the encoding string coupled with maintenance of non-random sequences selected by evolution over time (e.g., see [53,54] for a recent discussion of related ideas). This shift from low useful information content to high is a grayness at the origin of life. It should also be noted that recent work has defined individuals as entities with “temporal integrity” that “propagate information from their past into their futures” where information theory can then be used to separate the amount of information in any system from the information in the environment along a continuum [55]. Such an approach should prove useful for dealing with the grayness of information storage.

## 4. Metal Catalysis

The alternative competing perspective that is often used to examine the origin of life is the “metabolism-first” hypothesis, where life first arose through a series of self-sustaining chemical reactions. This corresponds to the metabolic subsystem of the chemoton model. In modern biology, metabolic processes are carried out by protein enzymes. Of these, between a third and half include metal ions in the active site for catalytic function [56]. These metalloenzymes are represented in all six Enzyme Commission classes [56]. Of particular interest are members of EC 1, the oxidoreductases, which carry out bioenergetic redox reactions and likely served as the earliest metabolic pathways in living systems. The primary metals present in oxidoreductases are Fe, Mo/W, or Cu [57]. The majority of biochemical pathways contain at least one metalloenzyme, including all major biogeochemical cycles [58]. The prevalent use of metal cofactors in modern enzymes, including ribozymes, as well as the ability for metals to catalyze reactions non-enzymatically, both environmentally and within cells, suggests that metals likely played an important role in the earliest metabolisms.

Mineral surfaces are known to concentrate organic molecules and promote chemical reactions [59]. Of these, metal sulfide surfaces are efficient environmental and industrial catalysts. Particularly notable is the work carried out by Huber and Wächtershäuser [60], showing that several iron sulfur minerals are able to spontaneously catalyze carbon fixation reactions, producing prebiotic molecules. Non-metals may also drive production of simple organic molecules as recent studies of radiolysis reactions driven by U, Th and K decay demonstrate production of simple organic acids such as acetate, formate and oxalate in addition to the more well known H_2_ production [22,61]. It is believed that the earliest forms of proto-life may have had metabolisms catalyzed by environmentally available metal-bearing nanominerals [8]. The shift from mineral surface catalysis to integrated metal clusters in proteins, or other polymer scaffolds, would permit evolutionary processes and result in increased catalytic rates, allowing for inhabitation of new environments [8,62].

An understanding of some of the earliest enzymes can be gained from phylogenetic reconstructions of the LUCA [10,63]. These analyses suggest that metal bioenergetic enzymes predate non-metal ones [63]. The use of organic cofactors in these redox enzymes is believed to have developed post-divergence and may have been driven by limited metal availability [63]. Metal inavailability is also offered as one of the original drivers for the adoption of protein enzymes, instead of exclusive reliance on non-enzymatic catalysts [64]. In phylogenetic studies, early enzyme cofactors that resemble metal sulfides, particularly Fe/S clusters, and oxyhydroxides, make these minerals favorable as pre-enzymatic catalysts [63,65]. Additionally, enzymes using Mo/W catalysts, due to their ability to perform two electron chemistry, appear to have arisen early in Earth history [63,65].

Of the various minerals that have been found to be structurally similar to enzyme active sites, greigite Fe^II^Fe^III^2S_4_, and violarite Fe^II^Ni_2_S_4_, are of particular interest [63]. These minerals are structurally similar to the active sites of CO dehydrogenase (CODH) and acetyl coenzyme-A synthase (ACS), respectively [63]. These enzymes are key components of the acetyl coenzyme-A pathway of carbon fixation, which is proposed to form organic carbon for the first living system in an autotrophic origin of life scenario similar to the earlier work of Wächtershäuser [66].

**Figure 4:**
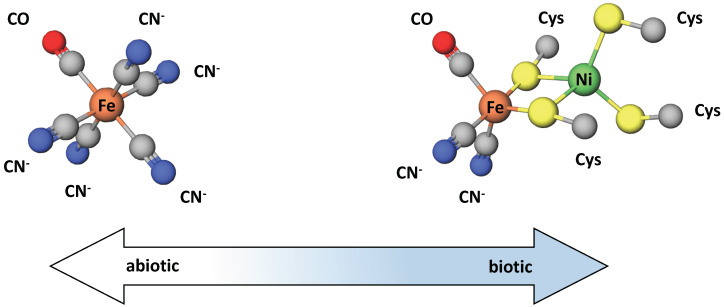
Grayness exists in the transition from catalysis via metal sulfides and metal oxides present in the abiotic environment to the use of similar structures in the active site of modern metalloenzymes. Here, we see the structure of an abiotic iron cyanocarbonyl complex, recently identified in some carbonaceous chondrites, and compare it to the active site structure of Fe-Ni hydrogenase. Note the active site ligands CN and CO, unusual in biology. Figure adapted from Ref. [68].

The similarity of the CODH and ACS enzyme metallocofactors with mineral structures has been discussed in support of a hydrothermal vent origin of life, where the physical and geochemical conditions at alkaline vents, including the prevalence of metal sulfide minerals, could have facilitated the rise of life [67]. However, other evidence suggests alternate chemical environments for early metallocofactors. A recent study of carbonaceous chondrite meteorites showed that most of the releasable cyanide, a substance of relevance in the synthesis of prebiotic molecules, was complexed with Fe and CO as [Fe^II^(CN)_5_(CO)]^3-^ and [Fe^II^(CN)_4_(CO)_2_]^2-^ [68]. Notably, these complexes are structurally similar to the active sites of Ni/Fe and Fe/Fe hydrogenases [68]. This similarity is shown in Figure 4. The fact that these enzymes have unusual inorganic cofactors, CN and CO, which are also proposed to be significant atmospheric gases in the prebiotic environment, has long been cited as evidence of an early origin.

Grayness exists in the inability to clearly demarcate between the biotic and abiotic. The similarities between metallocofactors and minerals in the environment illustrates the ambiguity that results from biochemistry’s stepwise emergence from geochemistry. Grayness also exists in the difficulty in distinguishing between the biotic and abiotic because of the co-evolution of Earth and life, beyond life’s origin. One such example of co-evolution is found in the metals used by organisms in their metalloenzymes.

The bioavailability of metals in the ocean has changed over Earth’s history [69]. This change corresponds to altered redox conditions following the Great Oxidation Event as well as periods of ocean euxinia, when the waters are anoxic and sulfidic. Metal usage is highly influenced by environmental availability [69]. Studies of metal binding motifs in enzymes show that the earliest evolved motifs were ambiguous, could incorporate more than one type of metal, or corresponded to metals that are soluble under reduced conditions, such as Fe or Mn [70]. Later evolving motifs corresponded to metals that became available after oxidative weathering freed them from sulfide minerals, or metals that are more soluble in an oxic environment, such as Cu or Zn [70]. The earliest proto-enzymes would have incorporated the metals most abundant in the surroundings. As the environment became more oxidized, the conservative nature of biology preserved higher usage of metals that had become less available. The relationship between elemental availability, particularly metals, and elemental usage by life is an area of growing research with implications for the origin of life on Earth, as well as life elsewhere [71].

The earliest proto-enzymes would be indistinguishable from the environment. This grayness would diminish as the environment changed and the organisms became more differentiated from their habitat, by retaining a biochemical dependence on metals that had diminished in availability. Once life evolved the ability to scavenge in instances of limitation, and store metals in instances of abundance, these organisms would have been able to explore and evolve within new environments. Under these conditions, life could be more clearly distinguished from its environmental background. Conversely, the ability to substitute metals that were rare with more plentiful metals, another survival strategy, would reduce the differences between cell and environment based on trace metal content. This would make life detection more difficult, increasing grayness. Such substitutions, when possible, have catalytic efficiency costs but are necessary adaptations to a dynamic environment for both ancient and modern life [72,73].

As can be seen, as a result of biochemistry’s origin in geochemistry, many of the progressive steps in development of a metabolic system would be ambiguous if viewed early in Earth’s history or on another world. This presents problems in terms of the discovery of life or proto-life elsewhere, as well as creates difficulty for determining whether a prebiotic environment would likely become a biotic system given adequate time.

## 5. Energy Currencies

All anabolic biochemical processes derive material from carboxylic acids produced in the citric acid cycle [74]. However, many carboxylic acids tend to react sluggishly to nucleophilic substitution reactions in water. To overcome energetic barriers, life employs group transfer reactions to create a more reactive intermediate. These “activated” intermediates are then readily converted to a product whose formation would otherwise be thermodynamically unfavorable from the initial starting compound. Life on Earth relies on phosphate compounds (e.g., ATP) or thioesters (e.g., acetyl-CoA) to lower energetic barriers for otherwise unfavorable reactions.

Thioester-based metabolism is hypothesized to have preceded phosphate-based metabolism [75]. This has been supported by recent computational work that demonstrates a potential thioester-dependent but phosphate-free network of metabolic reactions that includes the citric acid cycle [76,77]. Simple prebiotic thioesters could have plausibly been formed on early Earth [60,78,79,80,81], facilitated important prebiotic reactions such as amide bond formation, and exhibited autocatalytic behavior before the evolution of enzymes [82,83,84]. The evolution of a complex thiol group (e.g., coenzyme A, CoA) likely would have evolved as an enzymatic handle and could have been preceded by the pantetheine moiety of this compound [76,85]. Non-enzymatic pantetheine synthesis has been demonstrated under prebiotic conditions [86,87,88], and pantetheine thioesters could represent a “gray” area on the abiotic-to-biotic spectrum since only the distal end of this molecule is required for energy-transfer reactions.

While thioesters are universal in metabolism, phosphate-containing energy currencies (e.g., ATP) provide the energy to drive forward a majority of endergonic metabolic processes through phosphoryl-transfer reactions. The pyrophosphate moiety of ATP is a proposed predecessor to the more evolved adenosine-containing structure [89,90,91], and plausible prebiotic syntheses of pyrophosphate have been demonstrated [92,93,94,95]. However, the adjacent negative charges of pyrophosphate kinetically hinder nucleophilic attacks without selective catalysis and pyrophosphate therefore may not be the most ancestral mechanism of phosphoryl-group transfers [96]. Acetyl phosphate (or other mixed anhydrides) could potentially represent the earliest form of phosphate-based energy currencies [96,97] and an end-member on the abiotic-to-biotic spectrum. Acetyl phosphate could readily form and participate in non-enzymatic phosphorylation reactions [97] before the evolution of enzymes. Acetyl phosphate may further be the connection between thioester-based metabolism and phosphate-based metabolism, as is seen in the Wood-Ljungdahl pathway of carbon fixation, proposed to be present in the LUCA [10,98].

**Figure 5:**
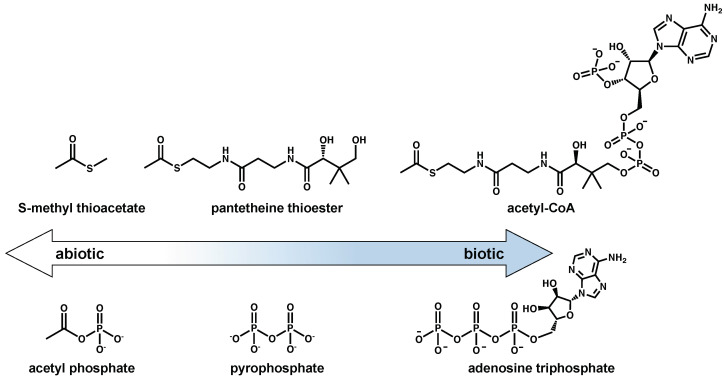
Possible evolution of thioester and phosphate-based energy currencies. A potential progression of small, prebiotically plausible energy currencies (S-methyl thioacetate, acetyl phosphate; (**left**) to those used in extant biochemistry (acetyl-CoA, adenosine triphosphate; (**right**) is shown. Grayness exists in between these endpoints (pantetheine thioester and pyrophosphate).

Both acetyl-CoA and ATP might represent examples of more evolved molecules with structures that indicate their utility for binding to enzymes. Early on in the abiotic-to-biotic transition, their roles of energy storage and transfer could have been filled by simpler molecules such as methyl thioacetate or acetyl phosphate. More complex energy currencies containing adenosine or pantetheine might have become more abundant in prebiotic chemical networks after the rise of proto-enzymatic machinery. Figure 5 shows this possible transition from simpler to more complex energy currencies.

## 6. Compartmentalization

Given their recalcitrance on geologic timescales, lipids are traditionally used as biosignatures in the sedimentary rock record and can be used to distinguish between the branches of life and reconstruct particular ecosystems [99,100,101,102]. Abiotic fatty acids are also easily differentiable from biotic lipids in their carbon number (odd versus even) and their carbon isotopes [103]. Lipids play a key role in modern cells, forming the necessary compartmentalization role that is theorized to be part of the trifecta of living systems.

Membrane forming lipids are generally two-tailed amphiphiles, and many of them contain phosphate headgroups. However, they differ between archaeal and bacterial/eukaryotic organisms: archaeal membranes use ether-linked hydrocarbons instead of ester linked, are of the opposite chirality in the glycerol backbone, and have branched hydrocarbons when compared to bacterial membranes [104]. These differences between lipids are much greater than the differences between the other large biomolecules (sugars, proteins, and nucleic acids) used by these phylogenetic branches, and helps us to better understand the grayness that could be found when looking for alternative encapsulation systems.

Membranes composed of lipids separate individuals to allow for selection, protect and co-localize metabolic and informational components, and drive energy production through chemiosmotic potential. This has given rise to the “lipid world” hypothesis that speculates that lipids could play a role in both informational and metabolic processes central in the development of life [105]. Specifically, short fatty acids can self-assemble into cell-like structures [106], encapsulate nucleic acids and protein [107], support metabolic and informational processes [108], and generate pH gradients [109]. These fatty acids are a product of prebiotic synthesis [110] and are also found in modern membranes, linking their alleged role throughout the origin and evolution of life.

Furthermore, fatty acids and/or other prebiotic amphiphiles, sometimes when mixed in specific combinations, have been found to self-assemble membranes under a wide range of environmental conditions [111,112]. Hydrophobic membrane-like structures are even found in hydrated meteorite extracts [113]. It has even been proposed that an oil slick would have covered Earth’s early oceans from prebiotic delivery of organics [114]. The ubiquity of the hydrophobic self-assembling organics in prebiotic sources makes a strong case for their presence throughout the universe and their diversity in extant biology is perhaps more revealing of their possible grayness as a biosignature.

Taking an even further step back, compartmentalization has also been proposed in more obscure structures for the origins of life [115]. Other colloids are often considered as primitive compartments, including oil droplets [116], aerosols [117], and coacervates [118]. The properties of these structures would be similar to membranes: the ability to grow and divide, and the interior and exterior are clearly defined and separated by a chemical phase. However, the material that makes these colloids would be very chemically different, giving an advantage for certain types of reactions (e.g., ribozyme activity can be favored in coacervates [119]). Inorganic structures have also been proposed as predecessors to lipid-based cells [120]. These mineral cells could be simple pores within rocks or even thin mineral membranes that can drive energy production [121,122]. There has been a large variety of structures proposed as alternative solutions to known biotic compartmentalization demonstrating an obvious grayness to this component of life.

## 7. Major Evolutionary Transitions

The grayness between non-living and living systems is not an idiosyncrasy within biology; there is ambiguity in many important evolutionary transitions, especially those associated with shifts in complexity or individuality. For example, suppose we consider a seemingly simple classification problem between unicellular and multicellular life, considered one of the major transitions in evolution [123]. Most would agree that a common *Escherichia coli* bacterium is unicellular while an elephant is multicellular, but it becomes trickier when one considers organisms such as *Dictyostelium discoideum*, the slime molds that alternate between unicellular and multicellular stages [124]. In the unicellular stage, they feed on bacteria, and when food runs out, some aggregate to form multicellular slugs that move and morph into fruiting bodies that disperse unicellular propagules to new environments. A further difficulty in the classification problem lies in distinguishing sociality from multicellularity, as many unicellular organisms live in communities and can exhibit emergent, population-level behaviors [125,126]. An analogous issue is distinguishing a population of multicellular organisms such as a herd of bison from a superorganism such as a bee colony. While both bison herds and bee colonies are groups of multicellular organisms, there are significant differences in their integration and degree of cooperation. Ultimately, even the word “organism”, which is a fundamental unit in biology, is nebulous and has been proposed to lie on a spectrum of organismality and be context-dependent [125,127]. Based on these issues, the origin of life is also likely to be an evolutionary transition with difficulties in distinction.

One reaction to the grayness within biology is to search for a conceptual framework that enables precise classification, while another reaction is to concede that such a framework does not exist and relegate the grayness to a set of exceptions. Yet, there is an alternative course that harnesses the grayness to improve our understanding of biological evolution and diversification. The grayness or spectrum of biological organization points to a fluidity in evolutionary transitions. In the example of distinguishing between unicellularity and multicellularity, there is a spectrum of sociality and cooperation that includes unicellular microbes that act multicellularly (e.g., through aggregative hunting or mobility as in *Myxococcus*) and multicellular organisms that act more unicellularly (e.g., as simple balls of undifferentiated cells that replicate as in *Gonium*)—in addition to the more canonical forms of unicellularity (e.g., *E. coli*) and multicellularity (e.g., elephants). Without the grayness, the evolution of multicellularity would reduce to a question of when does a unicellular organism evolve to be multicellular, as opposed to remaining unicellular. Embracing the grayness allows one to consider the vast array of alternative paths from unicellularity that result in populations exhibiting complex or interesting behaviors but that do not easily fit into the same category of multicellularity. Finally, the grayness may play an important role in shaping the evolution of different types of multicellularity, and we would never know this if we ignored it. Such roles are exemplified by the populations of microbes that facilitate (or inhibit) the appropriate development of complex multicellular organisms [128,129,130]. Embracing the power of a spectrum or “grayness” has been a major recent theme in astrobiology, analogous to the concept of dynamic habitability or the understanding that habitability is not a yes/no dichotomy but a gradient of multiparameter variation in time and space [131].

## 8. Conclusions

A grayness persists throughout biology. This is particularly true in life’s earliest transition from geochemistry to biochemistry. This grayness is associated with the degree of chemical disequilibrium. Life exists in disequilibrium from the environment and maintains this disequilibrium, using it to perform work. While the equilibrium state might be clearly abiotic, there is no inherent threshold to cross into the biological. Of these gray intermediate states, the distinction between proto-life at disequilibrium and a metastable mineral, for instance, is hard to discern. However, they might be compared to each other by “degree of aliveness”, which increases as biological innovations and optimizations are made [15]. Greater understanding of the spatial and temporal variability of disequilibria on Earth and on other bodies has merit for studies in the origin of life and astrobiology.

The definitiveness of a biosignature is inversely proportional to its accommodation of grayness—in other words, biosignatures that accommodate more diverse areas of biological grayness are less likely to result in a false negative detection. If we only consider life that is the most unambiguous and, therefore, the least gray, we may fail to recognize life that is very different or life that is very new. Not only would this result in missing a significant discovery, it also raises issues of planetary protection. Prebiotic chemicals with the potential for development into life may be disrupted, early cells may be denatured or even predated upon, and entire ecosystems, if present, may be affected. If our aim is detection of life early in its evolution, we must consider metrics with greater grayness, and therefore also more likelihood of false positives. With this abiotic-to-biotic spectrum, it is unlikely that there is a single metric which will allow for the unequivocal identification of life. Instead, we must use multiple agnostic approaches to life detection that are less reliant on preconceptions of terran biology. It should be noted that metrics that assign a continuum of values to characteristics of life inherently deal with grayness. For example, measures of how assembled a molecule is or of biological autonomy, individuality, and agency have all been developed recently and are relevant for assessing the degree to which a system possesses a particular biological property [53,54,55,132,133]. By considering multiple independent biosignature metrics, it is possible to create a theoretical framework in which the probability of a sample being of biological origin can be assessed. The practice of life detection must shift towards placement on a spectrum of certainty based on probability [1].

## Data Availability

This paper neither produced novel data nor analyzed existing data.
